# Cocoa polyphenols in brain health: BDNF/CREB-mediated mechanisms in depression, cognition, and the gut–brain axis

**DOI:** 10.3389/fnut.2026.1859145

**Published:** 2026-08-12

**Authors:** LePing Li, Henry H. Y. Tong

**Affiliations:** Faculty of Applied Sciences, Macao Polytechnic University, Macao, China

**Keywords:** brain health, cocoa polyphenols, cognitive function, depression, gut–brain axis, neurological disorders, spatial memory

## Abstract

Cocoa polyphenols have emerged as promising nutritional modulators of cerebral health through their multifaceted effects on neurobiological pathways. This review synthesizes contemporary clinical and preclinical evidence regarding their implications in depression, the gut-brain axis, neuroplasticity, and cognitive function across various neurological disorders and healthspan. Evidence derived from randomized controlled trials indicates that cocoa consumption may mitigate depressive symptoms and enhance mood states, potentially via the modulation of dopaminergic activity and emotional processing. Concurrently, cocoa polyphenols display prebiotic-like characteristics, altering the composition and diversity of gut microbiota, which may facilitate improvements in negative affect through the gut-brain axis. Nevertheless, the evidence pertaining to cognitive outcomes remains markedly inconsistent. While investigations utilizing animal models reliably indicate enhancements in spatial memory, hippocampal synaptic plasticity, neurogenesis, and equilibrium in oxidative stress, extensive human clinical trials yield either minimal or no substantial advantages in global cognitive function. Certain advancements are noted within specific areas, including executive function, alleviation of fatigue, and performance on tasks during stress-inducing scenarios. Mechanistically, cocoa polyphenols exhibit neuroprotective properties via the activation of Brain-derived neurotrophic factor/cAMP-response element binding protein signaling pathways, augmentation of cerebral blood circulation, antioxidant and anti-inflammatory effects, as well as the modulation of mitochondrial and apoptotic mechanisms. In summary, cocoa polyphenols present a potentially effective dietary approach for fostering brain health, particularly concerning mood regulation and neuroplasticity; however, additional rigorously designed research is essential to elucidate their cognitive effectiveness and clinical significance.

## 1 Introduction

Neurological disorders and depressive disorders represent a significant and intensifying global health challenge, influenced in part by the rise in life expectancy and demographic aging. Neurodegenerative conditions such as Alzheimer’s disease (AD), Parkinson’s disease (PD), and other associated ailments rank among the foremost contributors to disability-adjusted life years on a global scale (1). AD, in particular, is responsible for an estimated 60%–70% of dementia cases, with prevalence anticipated to escalate substantially in the forthcoming decades (2). Concurrently, major depressive disorder impacts over 280 million individuals worldwide and is a principal factor in years lived with disability (3). Notably, depression often coexists with neurodegenerative disorders, indicating potential shared pathophysiological mechanisms such as impaired neuroplasticity, chronic neuroinflammation, dysregulation of hypothalamic–pituitary–adrenal (HPA) axis activity, and oxidative stress (4, 5). At the molecular level, the pathophysiology of both neurological disorders and depressive states encompasses interrelated mechanisms such as mitochondrial dysfunction, excitotoxicity, compromised synaptic signaling, and diminished expression of neurotrophic factors, notably brain-derived neurotrophic factor (BDNF) (6, 7). BDNF serves as a pivotal regulator of neuronal survival, dendritic development, and synaptic plasticity, with its downregulation having been consistently documented in cases of depression and neurodegenerative diseases (8, 9). The transcription factor cAMP response element-binding protein (CREB) functions as a significant upstream regulator of BDNF expression, establishing a connection between extracellular stimuli and enduring alterations in neuronal functionality (10). Consequently, the disruption of the BDNF/CREB signaling pathway is regarded as a fundamental mechanism underlying cognitive deterioration, mood disorders, and deficits in learning and memory (11).

In this framework, there has been an increasing acknowledgment of the significance of dietary habits and nutritional intake as amenable determinants that impact cerebral health throughout an individual’s life span. Research in nutritional neuroscience has elucidated that dietary regimens abundant in fruits, vegetables, whole grains, and foods rich in polyphenols exemplified by the Mediterranean dietary pattern, are correlated with a diminished likelihood of experiencing cognitive deterioration and depressive disorders (12, 13). Bioactive dietary constituents, most notably polyphenols, invoke multifaceted effects on the central nervous system via mechanisms that encompass antioxidant, anti-inflammatory, and neuromodulatory pathways (14, 15). These compounds possess the capacity to influence intracellular signaling pathways (16), regulate the expression of genes, and modulate synaptic plasticity, thereby facilitating enhancements in cognitive performance and emotional regulation. An additional layer of intricacy emerges from the bidirectional communication between the gastrointestinal tract and the central nervous system, referred to as the gut–brain axis. This intricate system encompasses the integration of neural, endocrine, immune, and metabolic pathways, with the gut microbiota assuming a pivotal role (17). Dysbiosis of the gut microbiota has been associated with both depressive disorders and neurodegenerative pathologies, affecting neuroinflammation, neurotransmitter synthesis, and the integrity of the blood–brain barrier (BBB) (18). It is noteworthy that dietary polyphenols undergo extensive metabolism by gut microbiota into bioactive metabolites, which can subsequently modulate microbial composition and function, thereby establishing a reciprocal interaction that influences cerebral health (19, 20). This situates polyphenols as essential mediators of gut–brain communication.

Among foods characterized by a high polyphenolic content, cocoa (*Theobroma cacao*) has been recognized as an exceptionally promising candidate for neuroprotective strategies. Cocoa serves as an abundant reservoir of flavanols, encompassing compounds such as (−)-epicatechin, (+)-catechin, and oligomeric procyanidins, which demonstrate significant antioxidant capacity and superior bioavailability in comparison to other flavonoids (21). Empirical evidence indicates that these bioactive constituents possess the ability to traverse the BBB and accumulate within cerebral regions associated with cognitive functions, particularly in the hippocampus (22, 23). From a mechanistic perspective, cocoa-derived flavanols are capable of enhancing cerebral blood flow through nitric oxide-mediated vasodilation, improving endothelial function, and modulating neuroinflammatory processes (24). Beyond the vascular implications, cocoa polyphenols demonstrate direct neuromodulatory effects. Empirical investigations suggest that flavanols can activate crucial signaling cascades associated with synaptic plasticity, notably the ERK/cAMP response element-binding protein (CREB)/BDNF pathway, thus facilitating long-term potentiation (LTP), neurogenesis, and enhanced cognitive function (14). Epicatechin, in particular, has been evidenced to augment spatial memory and learning in animal models, in part due to elevated hippocampal BDNF expression and increased angiogenesis (25). Moreover, cocoa-derived constituents may modulate mood and alleviate depressive symptoms by influencing monoaminergic neurotransmission, diminishing neuroinflammation, and mitigating hyperactivity of the HPA axis (26).

Emerging clinical evidence substantiates these mechanistic findings, indicating that the consumption of cocoa flavanols may enhance cognitive performance, particularly in areas such as attention, executive function, and working memory, while also alleviating mental fatigue (27). Notwithstanding these encouraging observations, numerous gaps persist within the existing literature. The specific molecular mechanisms that associate cocoa polyphenols with neuroprotection especially through the interplay of gut microbiota-derived metabolites and intracellular signaling pathways such as BDNF/CREB, have yet to be comprehensively understood. Additionally, the degree to which these effects manifest across various neurological disorders and contribute to healthy aging (healthspan) necessitates a thorough integrative synthesis. Consequently, the aim of this review is to rigorously assess the existing body of evidence regarding cocoa polyphenols in relation to brain health, concentrating specifically on their implications for depression, the gut–brain axis, spatial memory and learning, as well as cognitive function. A particular focus is directed toward clarifying the role of BDNF/CREB signaling as a cohesive mechanistic pathway that underpins these effects in both pathological circumstances and the process of normal aging.

## 2 Cocoa polyphenols: composition, bioavailability, and metabolism

Cocoa-derived products represent some of the most abundant dietary sources of polyphenolic compounds, with a particular emphasis on flavonoids that belong to the flavan-3-ol subclass. These bioactive compounds have garnered considerable scholarly interest owing to their prospective neuroprotective, anti-inflammatory, and antioxidant characteristics, which are increasingly associated with the maintenance of brain health and the enhancement of cognitive function. The biological impacts of cocoa polyphenols are significantly modulated by their chemical composition, bioavailability, and metabolic pathways subsequent to consumption (28).

### 2.1 Major bioactive compounds

The principal bioactive components of cocoa polyphenols encompass monomeric flavan-3-ols, including (−)-epicatechin and (+)-catechin, in addition to oligomeric and polymeric procyanidins. Among these constituents, epicatechin emerges as the predominant and biologically active compound, demonstrating remarkable absorption efficiency and pronounced vascular and neuroprotective effects (29). Catechin, while occurring in lesser concentrations in comparison to epicatechin, plays a role in enhancing the overall antioxidant capacity of cocoa. Procyanidins, characterized by their oligomeric and polymeric structures derived from flavan-3-ol units, constitute a substantial fraction of cocoa polyphenols; however, their bioavailability is diminished due to their elevated molecular weight (30). These bioactive compounds exert their biological effects through a variety of mechanisms, which include the modulation of oxidative stress, the regulation of inflammation, and the alteration of intracellular signaling pathways such as the BDNF/CREB axis, a crucial pathway for synaptic plasticity and the processes underlying memory formation (14).

### 2.2 Absorption and metabolism

The bioavailability of cocoa polyphenols is significantly influenced by their specific chemical structure. Monomeric flavanols, with particular emphasis on epicatechin, exhibit efficient absorption in the small intestine, in contrast to larger procyanidins which are characterized by suboptimal absorption and undergo extensive metabolic processes (31). Upon absorption, both epicatechin and catechin engage in phase II metabolic processes within enterocytes and hepatocytes, culminating in the formation of conjugated metabolites such as glucuronides, sulfates, and methylated derivatives. These metabolites enter systemic circulation and are posited to mediate a multitude of systemic effects associated with cocoa consumption (32). The concentration of these metabolites in plasma typically reaches its zenith within 1–2 h’ post-ingestion, suggesting a rapid absorption and metabolic response. Nonetheless, their biological efficacy may diverge from that of the original compounds, thereby underscoring the necessity of accounting for metabolite profiles in mechanistic investigations (33).

### 2.3 Intestinal absorption

The intestinal uptake of cocoa flavanols predominantly transpires within the small intestine through mechanisms of passive diffusion, as well as potentially through carrier-mediated transport pathways. Monomeric flavanols, exemplified by epicatechin, exhibit a high degree of absorption efficiency, whereas oligomeric procyanidins demonstrate significant resistance to absorption, attributable to steric hindrance and diminished solubility (34). Elements that affect intestinal absorption encompass the food matrix, concomitant nutrients ingested, and individual differences in digestive physiology. For instance, the incorporation of dietary lipids may augment the solubility and absorption efficiency of lipophilic polyphenols, while proteins may interact with flavanols, thereby diminishing their bioavailability (35, 36). Moreover, the activity of intestinal enzymes and the prevailing pH conditions can significantly impact the stability and transformation of these compounds prior to their absorption, thereby contributing to the variability observed in systemic exposure.

### 2.4 Role of gut microbiota in biotransformation

A considerable fraction of cocoa-derived polyphenols, primarily procyanidins, arrives in the colon in an unaltered state, where they undergo extensive biotransformation facilitated by the gut microbiota. Microbial enzymatic activity deconstructs these intricate polyphenolic compounds into smaller phenolic acids and valerolactones, which exhibit enhanced bioavailability and may demonstrate significant biological implications (37). These microbial-derived metabolites have been demonstrated to traverse the BBB and may play a role in conferring neuroprotective effects, which include the modulation of neuroinflammation and the enhancement of synaptic plasticity. Furthermore, cocoa polyphenols possess the capacity to alter the composition and functionality of the gut microbiota, thereby fostering the proliferation of advantageous bacterial genera such as *Lactobacillus* and *Bifidobacterium* species (38). This reciprocal interaction between cocoa polyphenols and the gut microbiota underscores the significance of the gut–brain axis in mediating the neurological repercussions associated with cocoa consumption. Variations in individual microbiota composition may, therefore, substantially affect the effectiveness of cocoa polyphenols in interventions aimed at promoting brain health.

## 3 Cocoa polyphenols and depression symptoms

The relationship between cocoa polyphenols and depression has attracted increasing scientific attention because of the global prevalence of mood disorders and the limitations associated with current pharmacological therapies (39). Experimental evidence suggests that cocoa flavanols, particularly epicatechin, may influence several neurobiological pathways implicated in depression, including oxidative stress, neuroinflammation, mitochondrial dysfunction, and synaptic plasticity (14, 40). Preclinical studies further suggest that cocoa-derived polyphenols may modulate BDNF expression and CREB-related signaling pathways, thereby potentially contributing to neuronal resilience and mood regulation (14, 40). However, most mechanistic evidence has primarily been derived from experimental and animal studies, whereas clinical evidence in humans remains limited and heterogeneous. To avoid conceptual overlap, the potential role of cocoa polyphenols in modulating the gut–brain axis and gut microbiota is discussed separately in section “4 Cocoa polyphenols and the gut–brain axis” (41, 42).

### 3.1 Preclinical and clinical evidence

(A) Preclinical evidence

Preclinical investigations suggest that cocoa-derived compounds may influence depressive- and anxiety-related behaviors through modulation of oxidative stress, mitochondrial dysfunction, dopaminergic signaling, and neuroinflammatory pathways (43). In animal studies, chronic administration of elevated doses of 2-phenylethylamine (PEA) induced depression- and anxiety-like behaviors in Balb/c mice, including increased immobility in the forced swim test and reduced exploratory behavior in the elevated plus maze (43). These behavioral alterations were associated with depletion of striatal dopamine, increased hydroxyl radical production, and inhibition of mitochondrial complex I activity, suggesting a role for mitochondrial oxidative stress in mood-related disturbances (43). Nevertheless, oral administration of higher PEA doses in rats did not induce significant neurochemical or behavioral alterations, indicating that the translational relevance of these findings to normal dietary cocoa consumption in humans remains uncertain (43).

(B) Clinical evidence

Several clinical investigations have evaluated the effects of cocoa polyphenols or dark chocolate consumption on depressive symptoms, mood state, fatigue, and emotional wellbeing, although findings remain heterogeneous (44–50). A 4-week, double-blind, randomized, placebo-controlled trial investigated cocoa extract supplementation in 47 overweight or obese middle-aged individuals receiving an energy-restricted dietary regimen (44) (Table 1). Although depressive symptoms decreased in both groups, anxiety scores remained unchanged. Cocoa supplementation increased plasma homovanillic acid levels, which negatively correlated with depressive symptom changes, while dopamine fluctuations correlated with cocoa-derived metabolites (44). Another 12-week placebo-controlled trial involving 39 individuals with type 2 diabetes mellitus reported modest but statistically non-significant reductions in neuropathy scores and no significant improvements in glycemic parameters, quality of life, or somatosensory processing following cocoa supplementation (45). In menopausal women, consumption of 78% dark chocolate for 8 weeks significantly reduced depression scores compared with milk chocolate, whereas no significant changes were observed in sleep quality or anthropometric indices (46). Similarly, flavanol-rich cacao beverages significantly improved several negative mood parameters, including depression, fatigue, and anger, among middle-aged women with elevated oxidative stress levels (47). Conversely, a 10-day intervention study among university students demonstrated that fruit consumption resulted in more favorable psychological outcomes than chocolate/crisps intake, including lower anxiety, depression, emotional distress, fatigue, and cognitive difficulties (48). A randomized double-blind study further demonstrated that high-dose cocoa polyphenol beverages improved self-reported calmness and contentedness after 30 days, although no significant acute effects on mood or cognition were observed (49). Additionally, high cocoa liquor/polyphenol-rich chocolate improved fatigue, residual function, and mood in patients with chronic fatigue syndrome during an 8-week crossover intervention (50). Collectively, current clinical evidence suggests that cocoa polyphenols may exert modest beneficial effects on mood-related outcomes. However, interpretation of these findings should be cautious because available studies are limited by relatively small sample sizes, heterogeneous study populations, short intervention durations, and variability in cocoa composition and dosage (44–50).

**TABLE 1:** Effects of cocoa and chocolate on mood, depression, fatigue, and cognitive outcomes in human and animal studies.

Population and design	Intervention	Duration	Outcomes	Ref
47 overweight/obese adults (22 men, 25 women), double-blind, RCT	1.4 g/day cocoa extract (645 mg polyphenols) in a 15% energy-restricted diet vs. control	4 weeks	Depressive symptoms decreased in both groups; plasma homovanillic acid (HVA) increased 11.5% more in cocoa group; negative correlation between HVA change and depressive symptoms (β = −0.39, *P* = 0.029)	(44)
39 adults with type 2 diabetes, double-blind RCT	Cocoa capsules (50 mg polyphenols) vs. placebo	12 weeks	Toronto score and BEST score decreased in cocoa group, but differences vs. placebo not significant; no significant changes in glycemic profile, QoL, or H-reflex	(45)
60 menopausal women (45–65 y), triple-blind RCT	78% dark chocolate 12 g/day vs. milk chocolate 12 g/day	8 weeks	Depression scores significantly reduced in dark chocolate group (mean difference −2.3; *P* = 0.003; Cohen’s d = −0.54); no change in sleep quality or anthropometrics	(46)
60 women, aged 40–60 y, randomized, double-blind, placebo-controlled	Beverage with 240 mg cacao flavanols/day vs. placebo	8 weeks	Negative mood indicators (depression, fatigue, anger) and total mood disturbance decreased; positive mood (vigor) increased in cacao group (*P* < 0.05); no changes in fatigue scale or ANS activity	(47)
100 students (mean age 19 y; 27 M, 73 F), intervention study	Daily snack: fruit vs. chocolate/crisps	10 days	Fruit consumption reduced anxiety, depression, emotional distress, somatic symptoms, cognitive difficulties, and fatigue compared to chocolate/crisps	(48)
72 healthy middle-aged adults, randomized, double-blind, placebo-controlled	Dark chocolate drink with 0, 250, 500 mg polyphenols/day	30 days	High-dose cocoa polyphenols increased self-rated calmness and contentedness after 30 days; no effect on cognition or acute mood	(49)
10 subjects with CFS, double-blind, randomized crossover pilot study	High cocoa liquor/polyphenol-rich chocolate (HCL/PR) vs. iso-calorific low-polyphenol chocolate (CLF/LP)	8 weeks per arm, 2-week washout	Chalder Fatigue Scale improved with HCL/PR (*P* = 0.01) and worsened with CLF/LP (*P* = 0.03); residual function (London Handicap Scale) improved with HCL/PR and worsened with CLF/LP; Hospital Anxiety and Depression scores improved with HCL/PR	(50)
Balb/c mice, behavioral and neurochemical study	Chronic high-dose PEA (25–75 mg/kg i.p.)	Up to 7 days	PEA caused depression- and anxiety-like behaviors, motor deficits, dose-dependent striatal dopamine depletion, and hydroxyl radical generation; inhibited mitochondrial complex-I; no morphological changes in dopaminergic neurons	(43)

CFS, chronic fatigue syndrome; HCL/PR, high cocoa liquor/polyphenol-rich chocolate; CLF/LP, cocoa liquor-free/low polyphenol chocolate; HVA, homovanillic acid; CF, cocoa flavanols; HCF, high cocoa flavanol; LCF, low cocoa flavanol; PEA, 2-phenylethylamine; CFS, Chalder Fatigue Scale; ANS, Autonomic Nervous System; HPC, high-phenolic cocoa; LPC, low-phenolic cocoa; NTAD, non-transgenic Alzheimer’s disease; BDNF, brain-derived neurotrophic factor; NGF, nerve growth factor; RT, rotenone; TH, Tyrosine Hydroxylase; MAO, Monoamine Oxidase; RDD, rate-dependent depression; TOL, Tower of London Test.

### 3.2 Molecular mechanisms underlying cocoa effects on depression

Current evidence suggests that cocoa polyphenols may influence mood and neurocognitive function through several interconnected molecular pathways, although many of these mechanisms are primarily supported by preclinical studies (14, 40). Experimental findings indicate that cocoa flavanols may enhance BDNF expression and activate CREB signaling pathways, thereby potentially supporting synaptic plasticity, neurogenesis, and neuronal survival (14, 40). In addition, cocoa flavanols may improve cerebral blood flow and oxygen delivery, which could contribute to neuronal metabolism and cognitive processing. Experimental studies further suggest that cocoa-derived compounds possess antioxidant and anti-inflammatory properties through attenuation of reactive oxygen species generation, mitochondrial dysfunction, and pro-inflammatory cytokine production, including IL-6 (14, 40). In neurodegenerative experimental models, cocoa polyphenols have been reported to reduce amyloid beta-induced cytotoxicity, preserve neurite integrity, and modulate cellular survival pathways. Furthermore, combination therapies involving cocoa-derived compounds and nutraceutical agents such as vinpocetine, coenzyme Q10, and vitamin B have been associated with modulation of AKT/GSK-3β and Nrf2/HO-1 signaling pathways, as well as inhibition of apoptotic mediators including BAX and caspase-3. However, these findings are largely derived from experimental studies, and their direct clinical relevance remains to be established. Overall, available evidence indicates that cocoa polyphenols may exert neuroprotective and mood-related effects through antioxidant, anti-inflammatory, mitochondrial, and neurotrophic mechanisms. Nevertheless, additional translational and large-scale clinical studies are required to clarify the mechanistic relevance and clinical efficacy of cocoa polyphenols in depression.

## 4 Cocoa polyphenols and the gut–brain axis

### 4.1 Preclinical and clinical evidence

Clinical studies investigating the effects of cocoa polyphenols on the gut–brain axis have primarily focused on alterations in gut microbiota composition, gastrointestinal physiology, and brain activity (51, 52). One study demonstrated that daily intake of 85% dark chocolate (30 g/day) for 3 weeks improved negative affect in healthy adults, whereas 70% cocoa chocolate did not produce significant mood-related effects (51). Fecal 16S rRNA sequencing revealed that consumption of high-cocoa dark chocolate increased microbial diversity and *Blautia obeum* abundance while reducing *Faecalibacterium prausnitzii* levels (51). Importantly, reductions in negative affect negatively correlated with microbial diversity and *Blautia obeum* abundance, suggesting a potential association between cocoa-induced microbiota modulation and emotional state (51). Another randomized crossover trial evaluated the effects of 72% dark chocolate on gastrointestinal and cerebral function in healthy individuals (52). Although dark chocolate consumption did not significantly alter gastric emptying, it improved stool consistency and tended to prolong colonic transit time (52). PET-CT imaging further demonstrated increased glucose metabolism in visual, somatosensory, motor, and prefrontal cortical regions, suggesting altered cortical activity following cocoa consumption (52).

### 4.2 Molecular mechanisms underlying cocoa effects on gut–brain axis

**FIGURE 1:**
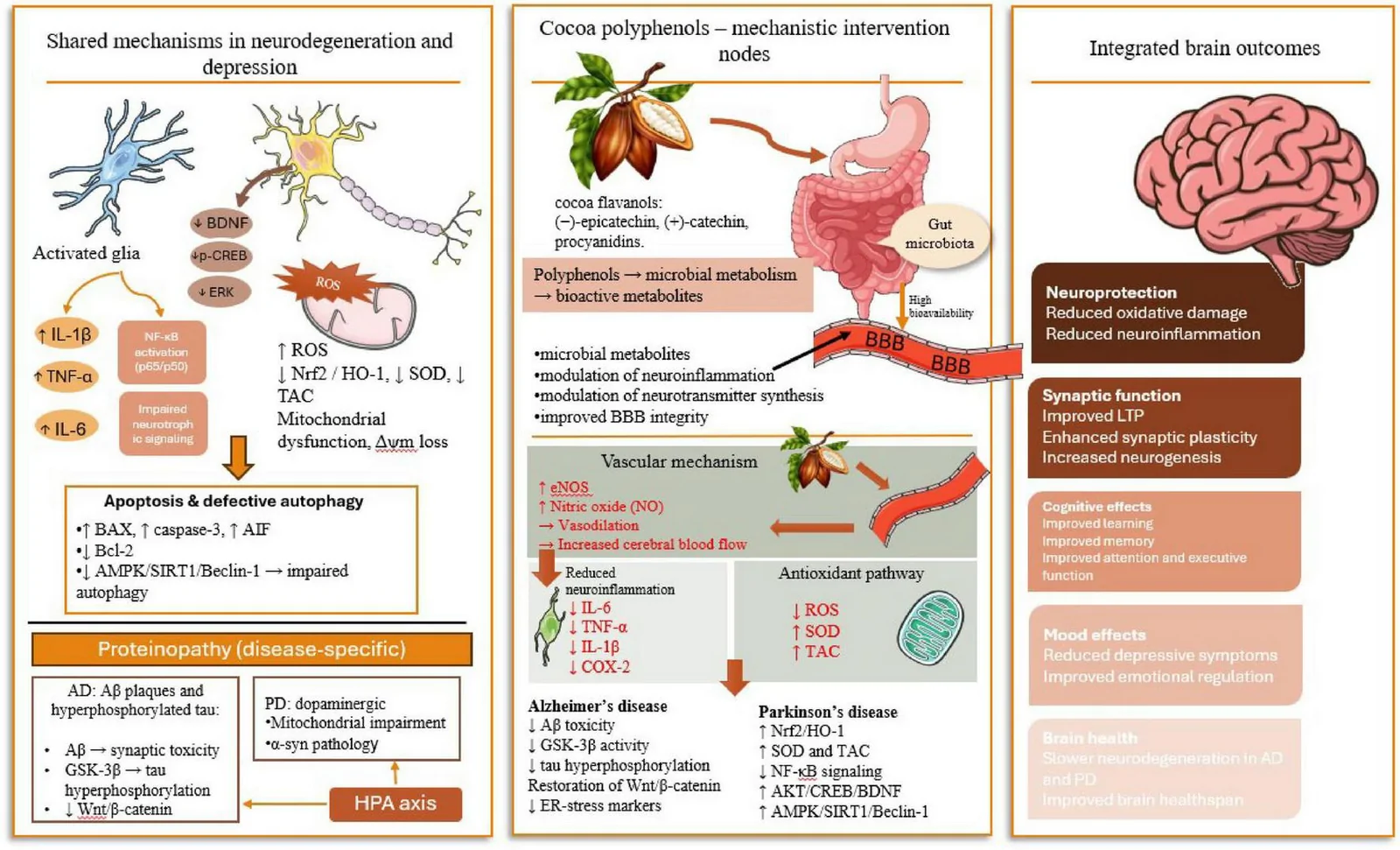
Mechanisms of neuroprotection by cocoa polyphenols. Neurodegenerative disorders and depression are associated with oxidative stress, neuroinflammation, impaired neurotrophic signaling, mitochondrial dysfunction, apoptosis, and defective autophagy. These alterations involve increased ROS production, activation of NF-κB–mediated inflammatory pathways (IL-6, TNF-α, IL-1β, COX-2), reduced ERK/CREB/BDNF signaling, and dysregulation of survival pathways including BAX, caspase-3, and Bcl-2. Disease-specific mechanisms such as amyloid-β toxicity, GSK-3β–mediated tau hyperphosphorylation, ER stress, and impaired Wnt/β-catenin signaling further contribute to neuronal damage. Cocoa polyphenols, particularly flavanols such as epicatechin, catechin, and procyanidins, exert neuroprotective effects through multiple mechanisms. These include activation of antioxidant defenses via the Nrf2/HO-1 pathway, inhibition of NF-κB–mediated inflammation, stimulation of ERK/AKT/CREB signaling leading to increased BDNF expression, and modulation of apoptosis and autophagy through Bcl-2, caspase-3, and AMPK/SIRT1/Beclin-1 pathways. Cocoa compounds also improve endothelial function and cerebral blood flow via nitric oxide production and influence the gut–brain axis through microbiota-derived metabolites. Together, these actions reduce oxidative stress and neuroinflammation, enhance synaptic plasticity and neurogenesis, and support cognitive function and mood regulation, thereby contributing to neuroprotection and potentially slowing the progression of neurodegenerative diseases.

Current evidence suggests that cocoa polyphenols may modulate the gut–brain axis through microbial, metabolic, gastrointestinal, and neural pathways (51, 52). High-cocoa dark chocolate consumption has been associated with increased gut microbial diversity and alterations in specific bacterial taxa, including increased *Blautia obeum* abundance (51). These microbial alterations may contribute to gut-derived metabolite production and subsequent neurochemical signaling involved in emotional regulation. In addition, cocoa polyphenols may influence gastrointestinal physiology by modulating stool consistency and colonic transit, potentially affecting enteroendocrine and neural signaling pathways (52). Neuroimaging findings demonstrating increased cortical glucose metabolism following cocoa intake further support the possibility that cocoa-derived compounds may influence gut–brain communication through neurophysiological mechanisms (52). However, current evidence linking cocoa-induced microbiota alterations with psychological outcomes remains preliminary, and causal relationships have not yet been conclusively established. Further mechanistic and longitudinal human studies are required to clarify the role of cocoa polyphenols in gut–brain axis regulation (51, 52) (Figure 1).

The metabolites derived from gut microbiota as a result of cocoa polyphenols are posited to exert influence on cognitive functions through various complementary mechanisms; nevertheless, the specific pathways through which these metabolites engage with the central nervous system and traverse the BBB have yet to be comprehensively elucidated. Following the metabolic processes conducted by gut microbiota, cocoa polyphenols are transformed into smaller phenolic acids (such as phenylacetic, phenylpropionic, and benzoic acid derivatives) that typically demonstrate enhanced systemic bioavailability in comparison to their parent compounds (53). It is plausible that these metabolites gain access to the brain through limited passive diffusion, contingent upon their physicochemical characteristics, or via transporter-mediated pathways at the BBB. Beyond the possibility of direct entry, it is probable that indirect mechanisms are significantly involved, encompassing the modulation of systemic inflammation, immune signaling, and the activation of gut–brain communication pathways, including the vagus nerve (17). Once introduced into systemic circulation, these metabolites and their subsequent signaling ramifications may exert influence on neuroinflammatory mechanisms, synaptic plasticity, and neurotrophic signaling cascades, encompassing BDNF-associated processes. Nonetheless, empirical evidence substantiating effective BBB penetration and targeted action within the central nervous system by cocoa-derived microbial metabolites is currently scarce. The prevailing comprehension is predominantly founded on indirect experimental findings, preclinical investigations, and metabolomic analyses, with the translational implications for human cognitive functioning remaining an area of ongoing research (17, 54). Additional mechanistic and clinical inquiries are imperative to elucidate these pathways and confirm causal relationships in human subjects.

## 5 Effects on neuroplasticity and synaptic function

**FIGURE 2:**
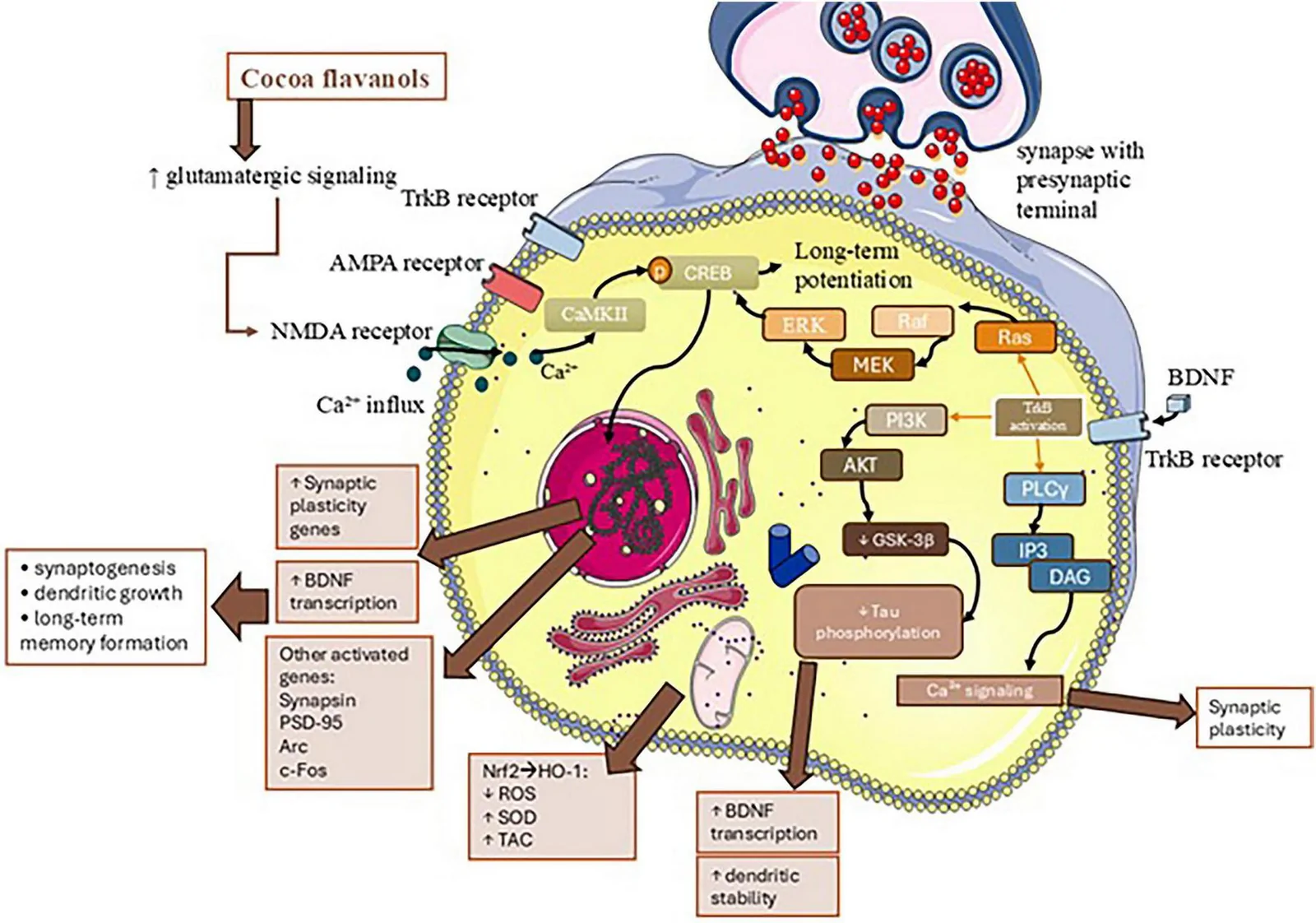
Molecular mechanisms by which cocoa flavanols enhance neuroplasticity and synaptic function. Cocoa flavanols, particularly epicatechin-rich polyphenols, enhance neuronal signaling pathways that regulate synaptic plasticity, dendritic growth, and memory formation. Cocoa flavanols increase glutamatergic neurotransmission and promote activation of postsynaptic NMDA and AMPA receptors, leading to Ca^2 +^ influx and activation of Ca^2 +^/calmodulin-dependent kinase II (CaMKII). This signaling cascade stimulates phosphorylation of the transcription factor CREB, which induces transcription of neuroplasticity-related genes including brain-derived neurotrophic factor (BDNF), synapsin, PSD-95, Arc, and c-Fos. BDNF released from neurons binds to its high-affinity receptor TrkB on the neuronal membrane, triggering multiple intracellular signaling pathways. Activation of the Ras–Raf–MEK–ERK (MAPK) cascade promotes CREB phosphorylation and strengthens long-term potentiation (LTP), a key cellular mechanism underlying learning and memory. In parallel, TrkB signaling activates the PI3K–AKT pathway, which inhibits glycogen synthase kinase-3β (GSK-3β), thereby reducing tau phosphorylation and contributing to neuronal stability and survival. TrkB activation also stimulates the PLCγ pathway, generating inositol-1,4,5-trisphosphate (IP3) and diacylglycerol (DAG), which amplify intracellular Ca^2 +^ signaling and further enhance synaptic plasticity. Cocoa flavanols additionally activate antioxidant defense mechanisms through the Nrf2–HO-1 pathway, leading to reduced reactive oxygen species (ROS) and increased activity of endogenous antioxidant systems such as superoxide dismutase (SOD) and total antioxidant capacity (TAC). These effects protect neuronal structures and mitochondria from oxidative stress. Together, these coordinated signaling pathways increase BDNF expression, strengthen synaptic transmission, promote synaptogenesis and dendritic growth, and ultimately support long-term memory formation and cognitive function.

Elucidating the role of cocoa polyphenols in neuroplasticity and synaptic function is essential for understanding their contribution to brain health and neural resilience (55). Neuroplasticity underlies learning, memory formation, and emotional regulation, and its impairment is a hallmark of neuropsychiatric and neurodegenerative disorders (56, 57). At the molecular level, cocoa flavanols modulate key synaptic signaling pathways, particularly the BDNF/CREB axis, which is critical for LTP, dendritic spine formation, and synaptic strengthening (Figure 2) (14, 58). In addition, cocoa polyphenols enhance cerebral blood flow and reduce neuroinflammatory signaling, thereby supporting synaptic integrity and neuronal communication (59). Bioactive metabolites derived from cocoa may also cross the BBB and influence neuronal signaling, although the extent of these effects in humans remains under investigation. Taken together, these molecular actions provide a mechanistic basis for the observed effects of cocoa on brain function; however, the strength of evidence varies significantly across experimental models and requires careful interpretation in translational terms.

### 5.1 Preclinical and clinical evidence

(A) Animal and preclinical evidence

Preclinical studies consistently demonstrate that cocoa-related interventions can modulate synaptic plasticity and cognitive performance in animal models. For example, combined dietary supplementation with chocolate, ω-3 polyunsaturated fatty acids, and probiotics in Wistar rats significantly improved spatial learning and memory in the Barnes maze. This effect was associated with hippocampal neuronal preservation in CA1 and CA3 regions and modulation of gut microbiota composition, suggesting indirect gut–brain axis involvement (60) (Table 2). In an Aβ1-42–induced AD rat model, cacao administration (0.5 g/kg/day) improved spatial and recognition memory while reducing oxidative stress and hippocampal neuronal degeneration (61). Similarly, another experimental study showed that cacao restored hippocampal LTP, improved antioxidant status, and reduced Aβ accumulation and synaptic dysfunction in AD-like pathology (62). Additional animal evidence further supports synaptic benefits: cocoa supplementation improved cholinergic transmission, reduced hippocampal cholinesterase activity, and enhanced spatial memory in an aged rat model of non-transgenic AD (63). Moreover, stress-induced synaptic impairments in rodents were partially reversed by dietary cocoa interventions, which restored synaptic strength and plasticity in the hippocampus (64). Conversely, not all animal studies demonstrate clear benefits. For instance, cocoa supplementation in combination with caloric restriction or treadmill exercise did not significantly alter BDNF levels or spatial memory, suggesting context-dependent effects (65).

**TABLE 2:** Effects of cocoa and related compounds on spatial memory and learning.

Model/participants	Intervention	Duration	Cognitive/neuroplasticity assessment	Findings	Ref
Male Wistar rats	Chocolate + ω3 PUFAs + probiotics	Not specified	Barnes maze (short- and long-term memory)	Combination improved spatial memory and learning; increased hippocampal CA1 and CA3 neuron numbers; no effect on brain weight, glucose, or epididymal tissue	(60)
Healthy young adults (*n* = 48)	Cocoa flavanols (415 mg), caffeine (215 mg), or combined	Acute, single session	Temporal attention, spatial attention, working memory tasks	No acute effects on working memory or attention from cocoa flavanols, caffeine, or combination	(66)
Rats, amyloid beta-induced AD model	Oral cacao (0.5 g/kg/day)	60 days	OF test, EPM test, NOR test, BM test, MWM test	Improved spatial memory and recognition memory; reduced anxiety; decreased oxidative stress and neuronal death in DG, CA1, and CA3	(61)
Rats, amyloid beta-induced AD model	Oral cacao (500 mg/kg/day)	2 months	Passive avoidance test, hippocampal LTP (fEPSPs, PS amplitudes), oxidative stress markers (TTG, MDA)	Improved passive avoidance memory, enhanced hippocampal LTP, modulated oxidative-antioxidative status, delayed Aβ plaque formation	(62)
Older adults, COSMOS-Clinic subcohort (*n* = 573, mean age 69.6 y)	Daily cocoa extract (500 mg flavanols, including 80 mg (−)-epicatechin)	2 years	Global cognition, episodic memory, executive function/attention	No significant overall benefit on global or domain-specific cognition; possible benefits in subgroup with poorer diet quality	(72)
Older adults with memory complaints (*n* = 259)	DHA-rich fish oil (1.1 g DHA + 0.4 g EPA) + flavanol-rich cocoa (500 mg/d)	12 months	Picture recognition task, other cognition and mood outcomes, structural neuroimaging	No cognitive improvement; some decline in executive function, alertness, reaction time variability; changes in plasma lipids and glucose confirmed intervention compliance	(73)
Healthy middle-aged adults	Dark chocolate intake	4 weeks	Fatigue assessment, executive function, memory, gray matter volume	Reduced mental and physical fatigue, enhanced executive function, memory, gray matter volume, and quality of life indirectly via fatigue reduction	(75)
Older adults, COSMOS-Mind RCT (*n* = 2262, mean age 73 y)	Cocoa extract (500 mg flavanols/day) vs. multivitamin-mineral (MVM)	3 years	Global cognition, memory, executive function composite scores	Cocoa extract showed no cognitive benefit; MVM supplementation improved global cognition, episodic memory, and executive function, especially in participants with cardiovascular disease	(74)
Rats under chronic isolation stress	Dark chocolate (varied dietary patterns: compulsory, optional, restricted)	Not specified	Hippocampal CA1 synaptic potency and plasticity (fEPSPs slope/amplitude, LTP), food intake, body weight	Compulsory and restricted DC reversed stress-induced impairments in hippocampal synaptic potency, plasticity, learning, and memory; all DC diets reduced food intake and body weight	(64)
Healthy young adults, NExp1 = 48, NExp2 = 32	Cocoa flavanols 415 mg	Acute, single administration	Visual working memory (passive maintenance and active updating)	No improvement in visual working memory recall, reaction time, or accuracy	(67)
Postmenopausal women (*n* = 140, aged 50–64)	10 g/day cocoa-rich chocolate (99%)	6 months	Attention, executive functions, verbal memory, working memory, phonological and category fluency	Slight improvement in cognitive flexibility and processing speed (Trail Making Test B); no changes in other cognitive variables	(68)
Healthy young adults (*n* = 98, aged 18–24)	35 g dark chocolate vs. 35 g white chocolate	Acute, 2 h post-consumption	Episodic verbal memory, mood	Dark chocolate improved episodic verbal memory relative to white chocolate; no mood effects	(69)
Healthy young adults, *n* = 18 (20–31 y)	Dark chocolate (DC) vs. white chocolate (WC) daily	30 days	Cognitive function: Stroop color word test, digital cancellation test; plasma NGF, BDNF, theobromine; prefrontal cerebral blood flow	DC increased NGF and theobromine, enhanced cognitive performance in both tests; effects persisted 3 weeks after end of intake	(70)
Healthy young men, *n* = 10	High CF (563 mg) vs. low CF (38 mg) beverage + moderate-intensity cycling	Acute, single session	Executive function (color-word Stroop), memory function (face-name matching)	HCF enhanced exercise-induced improvement in executive function but not memory function	(71)
Healthy adults, *n* = 32	Flavanol-rich vs. flavanol-poor chocolate	Acute, after 1 night of sleep deprivation	Working memory, psychomotor vigilance, SBP/DBP, flow-mediated dilation, pulse-wave velocity	Flavanol-rich chocolate preserved working memory, improved vascular function, mitigated sleep deprivation effects	(24)
Aged NTAD rats (17 mo)	Dark chocolate (70% cocoa, 4% polyphenols), 500 mg/kg/day	3 months	Cognitive performance: Barnes maze (spatial memory); hippocampal cholinesterase activity; histology (CA3 cell volume)	DC improved spatial memory, enhanced cholinergic activity, increased CA3 hippocampal cell volume, corrected metabolic disturbances	(63)
Wistar rats	Cocoa supplementation (2% w/w), treadmill exercise (1 h/day, 5x/week), caloric restriction	8 weeks	Morris maze test (spatial memory); oxidative stress (carbonylated proteins, ROS); BDNF levels	Exercise improved spatial memory and ROS tolerance; CR reduced free radicals; cocoa had no significant effect on ROS, BDNF, or memory	(65)
Female undergraduates, *n* = 96	Chocolate craving manipulation (24 h abstinence)	Acute	Working memory: digit span (phonological loop), Corsi blocks (visuospatial sketchpad), double span (central executive)	Chocolate craving selectively impaired visuospatial working memory (Corsi blocks), no effect on other components	(77)

AD, Alzheimer’s disease, Aβ, amyloid beta, BM, Barnes maze, CFS, Chalder Fatigue Scale, CE, cocoa extract, CLF/LP, cocoa liquor free/low polyphenols, COSMOS, COcoa Supplement and Multivitamin Outcomes Study, CR, caloric restriction, DC, dark chocolate, DHA, docosahexaenoic acid, EF, executive function, fEPSP, field excitatory postsynaptic potential, HCF, high cocoa flavanol, HCL/PR, high cocoa liquor/polyphenol rich chocolate, LCF, low cocoa flavanol, LTP, long-term potentiation, MVM, multivitamin-mineral, MWM, Morris water maze, NGF, nerve growth factor, NOR, novel object recognition, NTAD, non-transgenic Alzheimer’s disease, OM3FLAV, combined omega-3 PUFAs and cocoa flavanols, PEA, 2-phenylethylamine, PS, population spike, RDD, rate-dependent depression, ROS, reactive oxygen species, TAC, total antioxidant capacity, TOS, total oxidant status, TTG, total thiol group, UICV, unilateral intracerebroventricular, WM, working memory.

(B) Human clinical and experimental evidence

Human studies present more heterogeneous findings. Acute supplementation with cocoa flavanols (415 mg) with or without caffeine showed no significant effects on attention or working memory in young adults (66). Similarly, a controlled trial of cocoa flavanol intake in visual working memory tasks reported no improvement in cognitive accuracy or reaction time (67). In contrast, some interventional studies suggest domain-specific benefits. Daily consumption of 99% cocoa chocolate for 6 months improved cognitive flexibility and processing speed in postmenopausal women (68). Likewise, acute intake of dark chocolate improved verbal episodic memory in healthy young adults (69), while a 30-day intervention increased plasma NGF and was associated with improved cognitive performance and sustained effects after cessation (70). Furthermore, consumption of high-cocoa-flavanol beverages prior to moderate-intensity exercise improved executive function, although memory performance remained unchanged, suggesting selective cognitive effects of cocoa flavanols (71). However, large-scale randomized clinical trials provide more cautious conclusions. The COSMOS-Clinic trial (573 older adults, 2 years) found no significant effect of cocoa flavanol supplementation on global cognition or domain-specific cognitive outcomes (72). Similarly, the OM3FLAV trial combining omega-3 fatty acids and cocoa flavanols showed no cognitive benefit and even reported reductions in some executive functions and cortical volume (73). The COSMOS-Mind trial further confirmed the absence of significant effects of cocoa extract on global cognition, memory, or executive function in a large elderly cohort (74). Nevertheless, functional benefits unrelated to cognition were observed. For example, cocoa intake reduced fatigue and indirectly improved cognitive performance through enhanced vitality and emotional well-being (75). In addition, dietary pattern studies in rodents suggest that controlled cocoa intake may mitigate stress-induced synaptic dysfunction and support behavioral adaptation (64). Finally, experimental paradigms combining cocoa flavanols with exercise indicate that while cocoa may transiently enhance cerebral oxygenation, exercise-induced increases in cerebral perfusion and BDNF dominate cognitive outcomes, indicating a hierarchy of effects (76). Additional behavioral evidence suggests that chocolate-related neurocognitive effects may also involve motivational and rewards-related mechanisms. In a study of healthy female participants, chocolate craving induced selective impairment in visuospatial working memory without affecting phonological or executive domains, indicating that chocolate-associated motivational states may influence specific cognitive processes (77).

(C) Mechanistic bridging evidence (translational studies)

Translational and mechanistic studies provide additional insight into cocoa’s neurobiological actions. Cocoa flavanols increase cerebral oxygenation and NGF levels, which correlate with improved cognitive performance in healthy subjects (70). Moreover, *in vitro* AD models demonstrate that cocoa polyphenols activate BDNF signaling pathways and protect neurons from Aβ-induced toxicity through enhanced neurite stability and cellular survival mechanisms (78).

### 5.2 Molecular mechanisms underlying cocoa effects on neuroplasticity and synaptic function

At the molecular level, cocoa polyphenols regulate synaptic plasticity through multiple converging pathways. These include modulation of oxidative stress, reduction of lipid peroxidation, and suppression of neuroinflammatory mediators such as IL-6, TNF-α, and IL-1β. In parallel, cocoa compounds regulate apoptotic signaling by modulating BAX, Bcl-2, and caspase-3, thereby supporting neuronal survival. Furthermore, cocoa activates the AMPK/SIRT1/Beclin-1 pathway, promoting autophagic clearance and mitochondrial homeostasis. A central mechanism involves upregulation of BDNF and activation of CREB phosphorylation, which enhances synaptic plasticity, dendritic remodeling, and neurogenesis in the hippocampus (14). In addition, cocoa-induced improvement in endothelial function and cerebral perfusion contributes to enhanced neurovascular coupling, while modulation of neurotransmitter systems (dopaminergic, serotonergic, and cholinergic pathways) supports cognitive processing. These effects are further influenced by gut microbiota–derived metabolites, linking peripheral metabolic regulation with central synaptic function. Overall, cocoa exerts multi-target neurobiological actions that support synaptic integrity, although the magnitude of functional outcomes depends strongly on population characteristics, dosage, and baseline cognitive status.

## 6 Cocoa polyphenols and cognitive function

Investigating cocoa polyphenols in cognitive function is highly relevant in the context of aging and neurodegenerative disease prevalence (79). Cocoa flavanols, particularly epicatechin, have been associated with improvements in memory, attention, and executive function, primarily through vascular and neurotrophic mechanisms. Mechanistically, cocoa polyphenols modulate BDNF and CREB signaling pathways, which are essential for synaptic plasticity and neuronal survival (14). Additionally, their antioxidant and anti-inflammatory properties protect neurons from oxidative damage and age-related decline (27). The gut–brain axis also represents an important pathway through which cocoa metabolites may influence cognitive outcomes. Importantly, while section “5 Effects on neuroplasticity and synaptic function” focuses on synaptic and neuroplastic mechanisms, this section focuses specifically on functional cognitive outcomes and their translational evidence, thereby avoiding overlap.

### 6.1 Evidence from human and animal studies

(A) Preclinical and neurodegenerative disease models

Animal and mechanistic studies demonstrate that cocoa-related compounds exert neuroprotective effects in models of neurodegenerative disease. In a PD–related cellular model, a cocoa seed husk and guarana extract formulation enhanced cell viability, increased BDNF levels, and reduced mitochondrial dysfunction and neuroinflammatory signaling (80) (Table 3). In rotenone-induced PD animal models, cocoa especially in combination with vitamin B complex or other nutraceuticals, improved motor and cognitive performance and activated antioxidant (Nrf2/HO-1), neurotrophic (AKT/CREB/BDNF), and anti-apoptotic pathways (81). These findings highlight cocoa’s relevance not only to cognition but also to neurodegenerative resilience. In AD-related models, cocoa polyphenols improved hippocampal neurogenesis, enhanced object recognition memory, and increased BDNF expression (82). In aluminum-induced AD models, cocoa supplementation reversed tau hyperphosphorylation, oxidative stress, and synaptic dysfunction while restoring Wnt/β-catenin signaling and reducing neuronal apoptosis (58). Moreover, combined nutraceutical interventions containing cocoa, pomegranate, propolis, or VIN significantly improved neurotransmitter balance, antioxidant defense, and behavioral performance in rotenone-induced parkinsonism models (83).

**TABLE 3:** Cocoa and cognitive function: mechanistic insights via BDNF/CREB and neuroprotective pathways.

Model/participants	Intervention	Duration	Findings	Key mechanisms/signaling	Ref
SH-SY5Y cells, dPD-PBMCs, *Eisenia fetida* earthworms	Aqueous extract of cocoa seed husk + guarana (GuaCa, HCE3)	*In vitro*	↑ BDNF, ↑ cell viability, ↓ mitochondrial damage, ↓ IL-6	BDNF, mitochondrial protection, immunomodulation	(80)
Adult male Wistar rats (PD model)	L-DOPA/Carbidopa + CoQ10 + VIT B ± cocoa or VIN	19 days	Improved locomotor and cognitive outcomes, neurotransmitter balance, ↓ oxidative stress and inflammation, ↑ neurotrophic factors	Nrf2/HO-1, NF-κB, AMPK/SIRT-1/Beclin-1, AKT/GSK-3β/CREB/BDNF, apoptotic and autophagic pathways	(81)
C57BL/6JRj male and female mice	Standard diet vs. high-phenolic cocoa (HPC) or low-phenolic cocoa (LPC)	≥4 weeks	HPC ↑ BDNF, ↑ adult hippocampal neurogenesis, ↑ object recognition memory, minor effects on anxiety and locomotion	BDNF, hippocampal neurogenesis, neuroplasticity	(82)
Aluminum-induced AD rats	Cocoa alone or + VIN, EGCG, CoQ10, vitamins	5 weeks	↓ oxidative stress and inflammation, ↓ tau phosphorylation, ↑ BDNF, improved cognition and histology	Wnt3/β-Catenin/GSK-3β, Nrf2/HO-1, PERK/CHOP/Bcl-2, ER stress, apoptosis	(58)
60 healthy adults, 50–75 y	Cocoa powder, red berries, or combination	12 weeks	Improved executive function (TOL task), no significant change in BDNF or NGF-R	BDNF/NGF-R correlation with cognitive performance	(84)
Rotenone-induced PD rats	POM 150 mg/kg + VIN, propolis, or cocoa	20 days	Improved motor and cognitive performance, neurotransmitters, oxidative stress, inflammation, ↓ caspase-3	BDNF, GSK-3β, neurotransmitter modulation, antioxidant and anti-inflammatory pathways	(83)
12 healthy men	Acute 903 mg cocoa flavanols ± exercise	Single session	Improved cerebral oxygenation at rest, faster reaction time postexercise due to exercise, no effect on BDNF or executive function	Cerebral oxygenation, BDNF (not affected by CF), exercise-induced perfusion	(76)
Human AD *in vitro* model	Cocoa polyphenolic extract	*In vitro*	Neuroprotection against Aβ-induced neurotoxicity, prevented neurite dystrophy	BDNF survival pathway activation, antioxidant effects	(78)

BDNF, brain-derived neurotrophic factor; CF, cocoa flavanol; CoQ10, coenzyme Q10; CREB, cAMP response element-binding protein; EGCG, epigallocatechin-3-gallate; HPC, high-phenolic cocoa; LPC, low-phenolic cocoa; NGF-R, nerve growth factor receptor; RT, rotenone; VIN, vinpocetine; VIT B, vitamin B complex; AD, Alzheimer’s disease; EF, executive function; MF, memory function; PL, placebo; ΔHbO2, change in oxygenated hemoglobin; ΔHHb, change in deoxygenated hemoglobin; ΔHbtot, change in total hemoglobin; NTAD, non-transgenic Alzheimer’s disease model; PD, Parkinson’s disease.

(B) Human clinical evidence

Clinical studies in healthy and older populations show mixed outcomes. Some randomized trials report improvements in executive function, processing speed, and verbal memory following cocoa or flavanol intake (68, 69). Short-term studies also indicate increased NGF levels and improved cognitive performance correlated with vascular function (70). In addition, combined supplementation with cocoa flavanols and red berry anthocyanins improved executive performance in older adults, particularly in Tower of London task outcomes, although serum BDNF changes were not significant (84). However, multiple large-scale randomized controlled trials report no significant cognitive benefits of cocoa supplementation. The COSMOS-Clinic trial found no effect on global or domain-specific cognition (72), while OM3FLAV reported no cognitive improvement and even potential declines in executive function (73). The COSMOS-Mind trial similarly showed no cognitive benefit in a large elderly population (74). Nevertheless, secondary benefits such as reduced fatigue and improved emotional well-being may indirectly support cognitive function (75).

(C) Integrated translational evidence

Experimental human and animal studies suggest that cocoa may transiently enhance cerebral oxygenation and neurotrophic signaling; however, these effects do not consistently translate into measurable cognitive improvements (76). *In vitro* studies further confirm that cocoa polyphenols activate BDNF-dependent neuronal survival pathways and protect against Aβ-induced neurotoxicity (78).

### 6.2 Molecular mechanisms underlying cocoa effects on cognitive function

Cocoa polyphenols exert cognitive effects through multi-level biological pathways. At the cellular level, they reduce oxidative stress, inhibit pro-inflammatory cytokines (IL-6, TNF-α, IL-1β), and regulate mitochondrial apoptosis through modulation of BAX, caspase-3, and Bcl-2. Concurrently, activation of AMPK/SIRT1/Beclin-1 promotes autophagy and cellular homeostasis. A key mechanism involves activation of BDNF and CREB signaling, which enhances synaptic plasticity, neurogenesis, and neuronal survival. Cocoa also improves cerebral blood flow and endothelial function, thereby supporting neurovascular coupling. In addition, modulation of neurotransmitter systems and gut microbiota–derived metabolites contributes to broader cognitive regulation. Overall, cocoa exerts pleiotropic neurobiological effects; however, clinical outcomes depend on population heterogeneity, dose, duration, and baseline dietary context, explaining inconsistencies across trials.

Despite the accumulating evidence, considerable heterogeneity exists across studies regarding cocoa polyphenol composition, dosage, and formulation, including variability in flavanol content, cocoa percentage, and delivery matrix. This methodological inconsistency likely contributes to divergent findings across preclinical and clinical studies and limits direct comparability between trials. Future research should adopt standardized reporting of flavanol content (particularly epicatechin equivalents), clearly defined dosing regimens (mg/day), and harmonized intervention formulations to improve reproducibility and translational interpretation of results.

## 7 Limitations of current evidence

Despite the burgeoning interest in the potential advantages of cocoa polyphenols for cognitive health, several critical limitations persist. A predominant issue pertains to the relatively modest sample sizes in numerous clinical investigations, which may diminish statistical power and constrain the reliability and generalizability of the results. Furthermore, considerable heterogeneity is evident across studies in relation to intervention design, encompassing disparities in cocoa variety, polyphenol concentration, dosage, duration of supplementation, and participant characteristics such as age, health status, and baseline dietary habits. This variability complicates inter-study comparisons and may partially elucidate the inconsistent findings, particularly in relation to cognitive outcomes. Another significant limitation pertains to the absence of rigorously controlled mechanistic clinical investigations. While preclinical studies furnish compelling evidence for the engagement of pathways such as BDNF/CREB signaling, antioxidant defense mechanisms, and modulation of the gut microbiota, these mechanisms have not been adequately validated in human cohorts. Moreover, variations in the bioavailability and metabolism of cocoa polyphenols across individuals are frequently overlooked. Subsequent investigations should prioritize larger, standardized, and longitudinal clinical trials that integrate molecular, microbiome, and neuroimaging methodologies to enhance the understanding of the translational implications of cocoa polyphenols in relation to cerebral health.

## 8 Future perspectives

Future investigations regarding cocoa polyphenols and their implications for brain health ought to adopt more precise, integrative, and translational methodologies to elucidate their therapeutic capacities more effectively. A particularly promising avenue is the utilization of precision nutrition and personalized interventions. The individual differences in genetic makeup, metabolic pathways, gut microbiota profiles, and lifestyle determinants may exert a significant influence on the bioavailability and therapeutic efficacy of cocoa-derived polyphenols. Customizing interventions according to these individual attributes could improve responsiveness and optimize therapeutic outcomes, especially among populations predisposed to neurological disorders or cognitive decline. Furthermore, the incorporation of multi-omics technologies, encompassing microbiomics, metabolomics, transcriptomics, and proteomics, will be imperative to decipher the intricate mechanisms that underpin the effects of cocoa polyphenols. Such methodologies have the potential to yield a thorough comprehension of host-microbiome interactions, delineate bioactive metabolites, and elucidate the modulation of molecular pathways, such as BDNF/CREB signaling, across various physiological and pathological scenarios. The integration of these findings with sophisticated neuroimaging modalities may further clarify the nexus between molecular alterations and functional outcomes within the brain. Ultimately, there exists an imperative for meticulously structured, long-term randomized clinical trials featuring standardized interventions and explicitly defined endpoints. These investigations ought to consider factors such as dosage, duration, formulation, and participant characteristics to mitigate heterogeneity and enhance reproducibility. Such initiatives are indispensable for establishing causal relationships, validating clinical efficacy, and facilitating the translation of cocoa polyphenols into evidence-based nutritional strategies aimed at promoting brain health and preventing disease.

## 9 Conclusion

In conclusion, cocoa-derived polyphenols and catechins exert a diverse array of effects on neurocognitive health through intricate and interrelated molecular, cellular, and systemic pathways. The most robust evidence substantiates their advantageous role in the modulation of mood and depressive symptoms, likely facilitated by dopaminergic regulation, mitigation of oxidative stress, and enhancements in emotional processing capacities. Furthermore, nascent findings underscore the significance of the gut–brain axis, wherein cocoa-induced modifications in the composition and diversity of gut microbiota may play a role in diminishing negative affect and promoting psychological wellness. Conversely, the influence of cocoa polyphenols on cognitive functions, encompassing memory and learning, remains variable. Preclinical investigations consistently reveal enhancements in spatial memory, hippocampal neurogenesis, synaptic plasticity, alongside reductions in amyloid pathology and neuronal degeneration. These effects are frequently correlated with augmented antioxidant defenses and the modulation of LTP. Nevertheless, extensive randomized clinical investigations involving human subjects typically indicate negligible or no substantial enhancements in overall cognitive function. This discrepancy between preclinical and clinical findings may be attributed to several factors, including differences in metabolism and bioavailability of cocoa polyphenols between animals and humans, the use of substantially higher relative doses in experimental models, shorter intervention durations in clinical trials, and the greater biological and lifestyle heterogeneity present in human populations. In addition, animal models are often conducted under highly controlled conditions and may not fully replicate the complexity of human neurodegenerative and cognitive disorders. However, advantages may manifest in particular areas such as executive functioning, alleviation of fatigue, or in scenarios characterized by physiological stress. Current evidence suggests that cognitive benefits are more consistently observed in older adults, individuals with mild cognitive impairment or elevated physiological stress, and in studies employing moderate-to-high flavanol doses administered over longer intervention periods. However, variability in methodological design, participant characteristics, dosage regimens, intervention duration, and the bioavailability of cocoa-derived polyphenols continues to contribute to inconsistent findings across clinical studies. At the molecular level, activation of the BDNF/CREB signaling pathway has been widely proposed as an important mechanism underlying the neuroprotective and neuroplastic effects of cocoa-derived polyphenols, primarily based on preclinical and experimental evidence. In addition, modulation of related pathways such as Nrf2/HO-1, NF-κB, AMPK/SIRT1, and mitochondrial function has been reported in experimental models. However, direct evidence supporting a central role of BDNF/CREB signaling in mediating these effects in human studies remains limited and largely indirect. Therefore, this pathway should be interpreted as a promising but not yet fully confirmed mechanism in clinical contexts. Notwithstanding, translational links between these molecular findings and clinical outcomes are still insufficiently established. Subsequent investigations should emphasize the need for prolonged, rigorously controlled clinical trials that incorporate multi-omics approaches, neuroimaging techniques, and microbiome assessments to more effectively delineate the therapeutic promise of cocoa polyphenols in the context of neurological disorders and the facilitation of healthy cognitive aging.

## Author contribution

LL: Conceptualization, Data curation, Investigation, Methodology, Validation, Visualization, Writing – original draft, Writing – review & editing. HT: Conceptualization, Data curation, Investigation, Methodology, Supervision, Validation, Visualization, Writing – original draft, Writing – review & editing.
